# Rare Models: Use of a Novel Translational Model for Diffuse Alveolar Haemorrhage

**DOI:** 10.3390/ijms27104157

**Published:** 2026-05-07

**Authors:** Karan R. Chadda, Savithri Sathivelu, Susan K. Armstrong, Charlotte Edling, Kamalan Jeevaratnam

**Affiliations:** 1 Homerton College, University of Cambridge, Cambridge CB2 8PH, UK; 2 Institute of Inflammation and Ageing, University of Birmingham, Birmingham B15 2TT, UK; 3 Royal Free London NHS Foundation Trust, London NW3 2QG, UK; 4 Faculty of Health and Medical Sciences, University of Surrey, Guildford GU2 7XH, UK

**Keywords:** diffuse alveolar haemorrhage, pulmonary arterial hypertension, exercise-induced pulmonary haemorrhage, translational model, equine

## Abstract

Diffuse alveolar haemorrhage (DAH) is rare but associated with high morbidity and mortality. It is underpinned by mechanisms of endothelial dysfunction, inflammation, and vascular remodelling, yet its pathogenesis remains incompletely understood. Progress in translational research is currently limited by the absence of robust animal models of this condition. Equine exercise-induced pulmonary haemorrhage (EIPH), a common and progressive disorder in racehorses, may represent a novel and underutilised translational model for studying DAH, which can be a complication of or associated with other cardiopulmonary conditions, such as pulmonary arterial hypertension (PAH). EIPH occurs spontaneously in up to 95% of thoroughbreds after repeated strenuous exercise and is characterised by haemorrhage into the alveoli, capillary stress failure, inflammatory infiltration, and subsequent parenchymal remodelling. These processes closely parallel the vascular injury and immune activation observed in DAH. Horses further offer distinct advantages as models for human respiratory disease, including large lung size, long lifespan, and comparable pulmonary architecture. The regional distribution of haemorrhage in equine lungs additionally enables within-animal control studies. By leveraging the mechanistic overlaps between equine and human pulmonary pathology, EIPH provides a unique opportunity for biomarker discovery, mechanistic insight, and therapeutic testing in pulmonary vascular disease.

## 1. Introduction

Cardiovascular disease is a leading cause of global morbidity and mortality [1]. Accordingly, there has been significant clinical and research interest in advancing therapeutic strategies through the development of novel interventions [2]. This translational pipeline has relied heavily on mechanistic understanding of biological systems at the molecular and cellular level, before progressing findings into clinical studies [3]. A central challenge within this pipeline lies in the availability of appropriate animal models that recapitulate human disease mechanisms.

Importantly, translational research does not necessarily require identical replication of human disease, but rather models that accurately reflect relevant pathophysiological mechanisms. This principle underpins the widespread use of murine models for cardiac arrhythmia, despite significant interspecies differences in heart rate and electrophysiology [4], which have provided mechanistic insights applicable to human pathophysiology. The goal, therefore, is not to mimic clinical presentation precisely, but to model shared mechanistic pathways in a biologically relevant system.

The heterogeneity of cardiovascular, and other, diseases is increasingly understood through the concept of endotypes, which describe disease subtypes defined by distinct pathophysiological mechanisms rather than clinical presentation alone [5]. There is a growing understanding that using such a precision medicine approach is essential. By integrating biological and physiological data to determine endotypes, this method can identify reproducible patient subpopulations with different treatment responses [6].

In certain conditions, such as diffuse alveolar haemorrhage (DAH), defining endotypes remains challenging due to their relative rarity, diagnostic difficulties, poor access to human lung tissue, and lack of suitable preclinical models. Consequently, animal models are essential to investigate shared and distinct mechanisms of vascular injury, inflammation, and remodelling. However, while rodent models dominate preclinical research, they often fail to fully recapitulate the complex haemodynamics, lung structure, and chronic disease progression observed in humans [2].

This review explores equine exercise-induced pulmonary haemorrhage (EIPH) as a potential translational model to investigate DAH. A major barrier to translational progress is the lack of suitable preclinical models that replicate the mechanical, vascular, and inflammatory drivers of alveolar haemorrhage. This review therefore focuses on DAH as the primary disease of interest and evaluates EIPH in horses as a mechanistically relevant translational model of alveolar bleeding. While traditionally studied within veterinary medicine due to its high prevalence in athletic horses, the mechanistic overlap between EIPH and human pulmonary vascular pathology suggests underutilised potential for human-focused translational research.

## 2. Diffuse Alveolar Haemorrhage

Diffuse alveolar haemorrhage (DAH) is a life-threatening condition arising from alveolar blood accumulation, often manifesting with rapid-onset respiratory deterioration [7]. In DAH, red blood cells extravasate from the pulmonary microvasculature into alveoli, impairing gas exchange, precipitating hypoxaemia, and potentially evolving into acute respiratory distress syndrome (ARDS) [8,9]. The clinical presentation is typically acute or subacute, with symptoms evolving over hours to days [10]. The prognosis is poor, with recurrent episodes associated with interstitial fibrosis [11,12], and in-hospital mortality between 20 and 100% [13]. While considered rare, DAH accounts for ~12% of intensive care admissions in patients with autoimmune diseases [14].

The presenting symptoms, including haemoptysis, dyspnoea, cough, and fever, are non-specific and have variable severity, which delays diagnosis [15]. Although haemoptysis is classically associated, it is absent in up to one-third of cases, particularly when bleeding is diffuse and diluted in the alveolar volume. Thus, absence of overt haemoptysis should not exclude DAH from the differential diagnosis [10]. The majority of DAH cases are associated with immune-mediated capillaritis, including ANCA-associated vasculitis, anti-glomerular basement membrane (anti-GBM) disease, systemic lupus erythematosus (SLE), or other systemic autoimmune conditions. Nonimmune causes include coagulopathies, drug toxicity, inhaled toxins, infectious insults, mitral valve disease, or pulmonary venous hypertension among others [16].

From a diagnostic standpoint, DAH is inherently challenging because its signs and radiographic findings are non-specific. The bilateral pulmonary infiltrates or ground-glass opacities on imaging include differentials such as infection or oedema. Definitive diagnosis through bronchoalveolar lavage and observation of haemosiderin-laden macrophages with biopsy are both invasive [9].

Importantly, DAH represents a syndrome of alveolar barrier failure, rather than a single disease entity, with diverse triggers including immune-mediated capillaritis, coagulopathy, and haemodynamic stress. This heterogeneity highlights the need for mechanism-focused models that isolate specific drivers of alveolar haemorrhage, such as capillary stress failure and inflammatory injury. Given its rarity and heterogeneity, DAH continues to suffer from a paucity of prospective trials or mechanistic studies, reinforcing the urgent need for improved animal models and translational research.

## 3. Equine Exercise-Induced Pulmonary Haemorrhage (EIPH) as an Animal Model for DAH—An Unfulfilled Opportunity

For DAH, it is difficult to access human lung tissue samples to investigate the pathophysiological mechanisms and establish biomarkers. Furthermore, there are limited animal models for this condition, and to our knowledge, there are no routinely used animal models with these diseases spontaneously occurring.

We propose that equine exercise-induced pulmonary haemorrhage (EIPH) shares many common features with both DAH, and thereby represents a novel translational model. EIPH is associated with strenuous exercise in horses and essentially any horse working at speeds greater than 240 m/min is at risk of EIPH [17,18,19,20]. EIPH is thought to occur in more than 50–75% of thoroughbreds (TBs) post-race and prevalence may increase to >95% after repeated runs [21,22,23]. A currently incurable condition, EIPH is occasionally associated with sudden death on racetracks [24,25,26,27]. It is considered a progressive condition [28,29,30,31]. Similar pathophysiological mechanisms occur in EIPH to DAH, including changes in intravascular pressures (capillary, positive), extravascular pressures (alveolar, negative), disruption of the blood–gas barrier (strength/fragility) and alteration of lung parenchyma (reduced compliance/fibrosis) [32,33,34,35]. Genetic predisposition linked to cell stiffness may further contribute to susceptibility [36]. An inflammatory response also contributes to disease progression [37,38].

Equine EIPH is a well-suited model to study DAH pathophysiological mechanisms due to its spontaneous occurrence and the horse’s long lifespan and large dimensions. The pulmonary systems of horses and humans share key similarities, including lung structure, large capacities, mucociliary clearance, immune responses, and development of certain conditions, such as asthma and progressive fibrosing lung diseases [39,40].

The higher absolute pulmonary arterial pressures in horses should not be viewed as pathologic but leveraged to investigate how sustained vascular stress induces microvascular damage, remodelling, and impaired compliance, mechanisms central to DAH pathogenesis. In horses, blood flow increases from the ventrodorsal regions and is highest towards the dorsocaudal region, which is most impacted by EIPH, meaning the ventrocranial lung fields are almost entirely unaffected [31]. This provides an excellent means to study lung tissue of EIPH-positive horses by histopathology and proteotranscriptomic profiling as they can act as their own study controls.

## 4. Other Relevant Human Clinical Conditions

While EIPH represents a compelling model of alveolar bleeding seen in DAH, offering opportunities to investigate vascular stress, barrier failure, and early remodelling processes further, EIPH may also be a relevant model for other human conditions.

In progressive lung diseases like idiopathic pulmonary fibrosis (IPF), the upregulation of certain miRNAs is often linked to ongoing lung remodelling [41], a process also critical to the pathology of EIPH. Various miRNAs involved in EIPH progression have been characterised in human lung disease research. For example, fibrosis-linked miRNAs, including miR-21, miR-29, and miR-200, are implicated in the vascular remodelling seen in pulmonary hypertension and other conditions [42,43]. Conversely, molecules like miR-150 have demonstrated a protective effect, mitigating hypoxia-induced fibrosis and vascular remodelling in pulmonary hypertension [43]. Given that candidate genes for hypoxic angiogenesis are implicated in EIPH horses [44], the potential for miR-150 is significant; experimental models show that its upregulation suppresses disease-promoting pathways and restores normal vascular architecture, highlighting its utility for both diagnosis and future therapeutic intervention [43].

Pulmonary arterial hypertension (PAH) is a progressive syndrome characterised by remodelling of the pulmonary vasculature and a sustained increase in pulmonary vascular load [45]. It is hemodynamically defined by a mean pulmonary arterial pressure greater than 20 mmHg at rest, as assessed by right heart catheterization [46]. A common pathophysiological feature of PAH is vasculature remodelling from endothelial dysfunction and infiltration of inflammatory cells, causing luminal narrowing or complete obliteration of small vessels, and raising pulmonary vascular pressure [47,48]. There may be a role of inflammation in the pathogenesis of PAH based on the accumulation of perivascular inflammatory cells and circulating levels of certain cytokines [48]. Although DAH and PAH differ in acuity and aetiology, they share several mechanistic processes: endothelial dysfunction and barrier failure, inflammatory activation, vascular remodelling and fibrosis, and haemodynamic stress [16,45]. Thus, a better mechanistic understanding of EIPH may have translational relevance for both DAH and PAH, due to some of the overlapping pathological features.

## 5. Potential Future Directions and Limitations of the Model

The significant but underexplored translational potential of using equine exercise-induced pulmonary haemorrhage (EIPH) as a model for human diffuse alveolar haemorrhage (DAH) and other human conditions could guide future research directions. By conducting comparative molecular profiling studies, researchers can identify shared biomarkers and underlying pathways between EIPH and DAH. This could help define distinct endotypes of EIPH that may highlight parallel mechanistic subtypes in human DAH, thereby facilitating the development of a more precise, individualised medicine approach. Furthermore, histopathological mapping of affected versus unaffected lung regions in horses could provide insights into vascular stress thresholds and the patterns of remodelling, which are central to the pathogenesis of DAH. The large size of the horse also makes it an ideal subject for therapeutic trials, allowing for the testing of inhaled or systemic agents in a way that bridges the gap between small-animal experiments and human clinical trials.

There are several limitations of equine models to consider. It is important to acknowledge that while EIPH may model mechanisms of alveolar haemorrhage seen in DAH, it will likely not encapsulate the full spectrum of DAH pathophysiology due to differences in human and equine physiology. For example, equine pulmonary artery pressure is higher than in humans, reaching close to 100 mmHg at a gallop [49]. The equine model is well established for the study of human asthma, although horses tend to develop pulmonary neutrophilia instead of eosinophilic inflammation in severe cases which differs from humans [50]. As eosinophils have not been shown to have a significant role in DAH pathogenesis, this would not invalidate the equine EIPH model. Instead, there is evidence of neutrophilic inflammation in DAH pathogenesis [11]. Other limitations include substantial financial costs related to acquisition, maintenance, and housing, alongside ethical considerations. In addition, the relatively limited availability of species-specific reagents, incomplete genomic and tissue-level characterisation, prolonged maturation periods, and the large physical size of horses can constrain the scope and feasibility of certain experimental approaches [51].

## 6. Conclusions

Diffuse alveolar haemorrhage is associated with significant morbidity and mortality, with limited understanding of pathogenesis, and a lack of reliable biomarkers. Progress in these fields is hindered by the absence of robust preclinical models that replicate disease mechanisms. While no animal model can replicate these conditions in entirety, equine EIPH represents a compelling translational model. With spontaneous onset, progressive pathology, and mechanistic overlap with DAH, it offers unique opportunities for mechanistic, biomarker, and therapeutic research for these conditions that warrants exploration and validation in future studies.

## Data Availability

No new data were created or analyzed in this study. Data Sharing is not applicable.
